# Petrosamine isolated from marine sponge *Petrosia* sp. demonstrates protection against neurotoxicity in vitro and in vivo

**DOI:** 10.1007/s13659-024-00439-x

**Published:** 2024-02-22

**Authors:** Joana Ribeiro, Henrique Araújo-Silva, Mário Fernandes, Joilna Alves da Silva, Francisco das Chagas L. Pinto, Otília Deusdenia L. Pessoa, Hélcio Silva Santos, Jane Eire Silva Alencar de Menezes, Andreia C. Gomes

**Affiliations:** 1 CBMA (Centre of Molecular and Environmental Biology) / Aquatic Research Network (ARNET) Associate Laboratory, Department of Biology, University of Minho, Campus de Gualtar, 4710-057 Braga, Portugal; 2 Program in Natural Sciences, Natural Products Chemistry Laboratory, State University of Ceará, Fortaleza, Ceará Brazil; 3 Department of Organic and Inorganic Chemistry, Science Center, Federal University of Ceará, Fortaleza, Ceará Brazil

**Keywords:** Petrosamine, *Petrosia* sp., Neuroprotection, Aluminium-induced neurotoxicity

## Abstract

According to The World Alzheimer Report 2023 by Alzheimer’s Disease International (ADI) estimates that 33 to 38.5 million people worldwide suffer from Alzheimer’s Disease (AD). A crucial hallmark associated with this disease is associated with the deficiency of the brain neurotransmitter acetylcholine, due to an affected acetylcholinesterase (AChE) activity. Marine organisms synthesize several classes of compounds, some of which exhibit significant AChE inhibition, such as petrosamine, a coloured pyridoacridine alkaloid. The aim of this work was to characterize the activity of petrosamine isolated for the first time from a Brazilian marine sponge, using two neurotoxicity models with aluminium chloride, as exposure to aluminium is associated with the development of neurodegenerative diseases. The in vitro model was based in a neuroblastoma cell line and the in vivo model exploited the potential of zebrafish (*Danio rerio*) embryos in mimicking hallmarks of AD. To our knowledge, this is the first report on petrosamine’s activity over these parameters, either in vitro or in vivo, in order to characterize its full potential for tackling neurotoxicity.

**Graphical Abstract:**

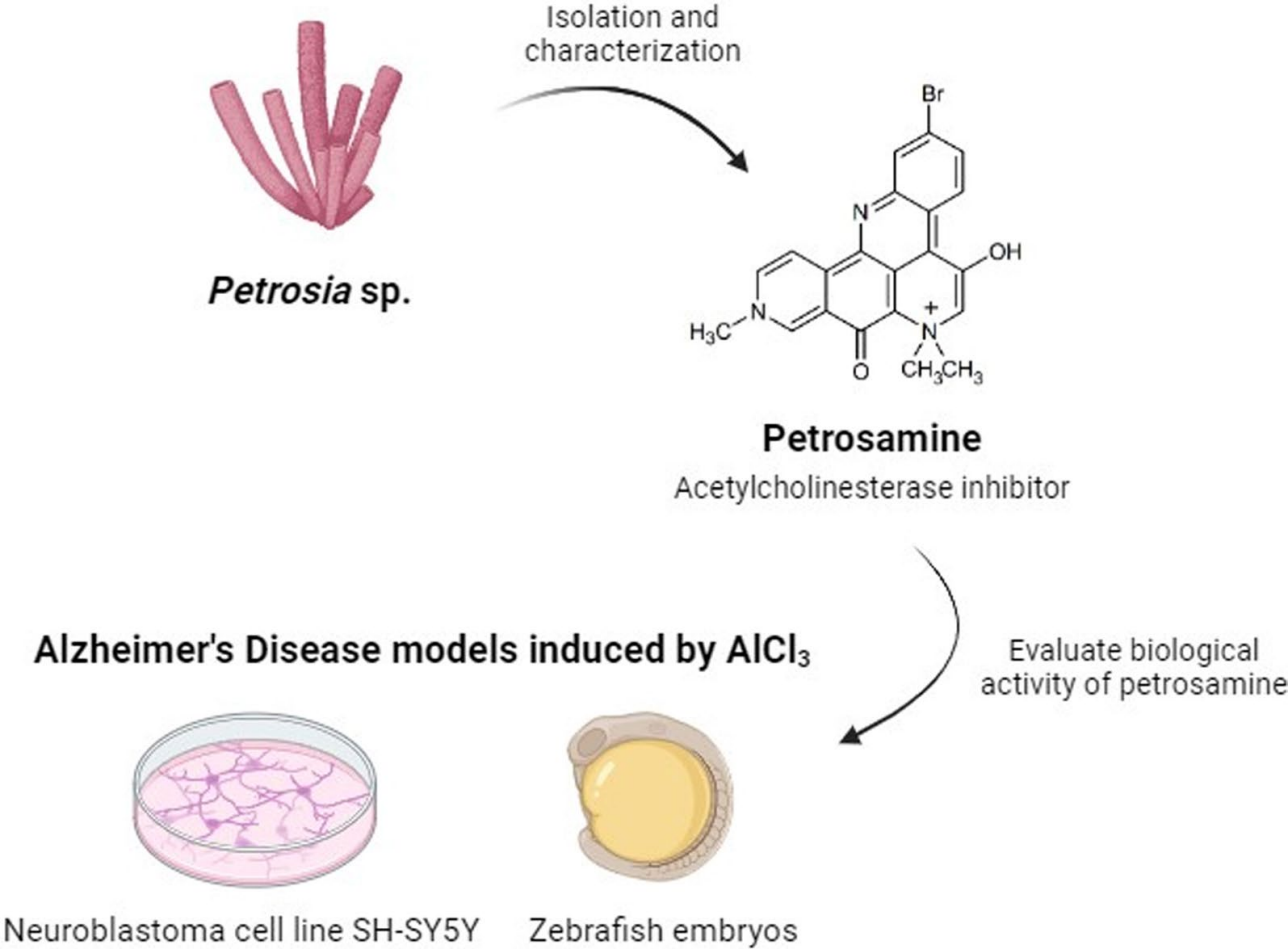

**Supplementary Information:**

The online version contains supplementary material available at 10.1007/s13659-024-00439-x.

## Introduction

Neurodegenerative diseases (NDDs) are a heterogeneous group of neurological disorders, characterized by the progressive loss of neurons in the central nervous system (CNS) [1, 2]. Alzheimer’s Disease (AD) is an example of a NDDs, adversely affecting the lives of millions of people worldwide, with 7.24 million cases registered in 2019 alone ([3], pp. 1990–2019). The pathophysiological development of AD is complex and multifactorial, and the exact mechanism remains unclear. A crucial hallmark associated with this disease is associated with the deficiency of the brain neurotransmitter acetylcholine (ACh), due to an affected acetylcholinesterase (AChE) activity, a key enzyme in the cholinergic nervous system. The inhibition of AChE prevents the breakdown of ACh into acetate and choline and subsequently increases its concentration and duration of action, which is beneficial for AD patients [4]. In fact, the enhancement of the cholinergic neurotransmission represents a widely used approach in the treatment of symptoms of mild and moderate stages of AD [5].The brain is one of the most complex organs in the human body due to its unique structure and function. The development of effective therapeutics to treat AD is still a clinical challenge that has not been achieved so far, owing to a limited understanding of the disease. Consequently, model systems that can reproduce the pathological and biochemical characteristics of AD are very important to better study this pathology and, therefore, improve translational value of novel therapeutics [6].

Marine organisms have their own defense mechanisms, by synthesizing several classes of compounds, such as alkaloids, in order to defend themselves. Consequently, the identification of marine natural products rich in diverse biological activities are a need for the AD treatment [7]. Pyridoacridine alkaloids constitute one of the largest chemical families of marine-derived alkaloids, usually isolated from marine sponges, tunicates, anemone, and molluscs. There has been great interest on the biological activity of these compounds, confirming this family of alkaloids as a source of new lead structures for drug development [8, 9] including as possible treatments for AD, since it has also been reported that these compounds can inhibit AChE activity [10]. Petrosamine is a coloured pyridoacridine alkaloid, isolated from the sponge *Petrosia* sp., with a potent AChE inhibition. This compound has an AChE inhibitory activity about six times the potency of the galanthamine, a current AChE inhibitor used in the treatment of AD [9]. Petrosamine shows strong AChE inhibitory activity due to their molecular structure, that greatly interacts with AChE. Structurally, petrosamine is a planar heterocyclic complex nitrogen-containing system and is a derivative from two amino acids, tryptophan and tyrosine. The most important interaction between petrosamine-AChE is related the interaction of the positively-charged nitrogen of petrosamine [7, 11].

The aim of this work was to characterize the activity of petrosamine isolated for the first time from a Brazilian sponge *Petrosia* sp., using two AD models, one based in a neuroblastoma cell line and another using zebrafish embryos (*Danio rerio*), that mimic hallmarks of AD. For the establishment of these neurotoxicity models, aluminium chloride (AlCl_3_) was used. Aluminium (Al) exposure is linked to the progression of neurodegenerative diseases and many studies have demonstrated that this metal crosses the BBB, accumulating in a semipermanent manner [12, 13]. Several molecular mechanisms are affected by Al, such as protein accumulation, formation of reactive oxygen species (ROS) and lipid peroxidation, which mimics symptoms of AD pathogenesis [14]. To our knowledge, petrosamine’s activity on these parameters has never been studied before, either in vitro or in vivo, to identify properties of this compound against Al-induced neurotoxicity.

## Results, discussion and conclusion

### Extraction and isolation

**Fig. 1:**
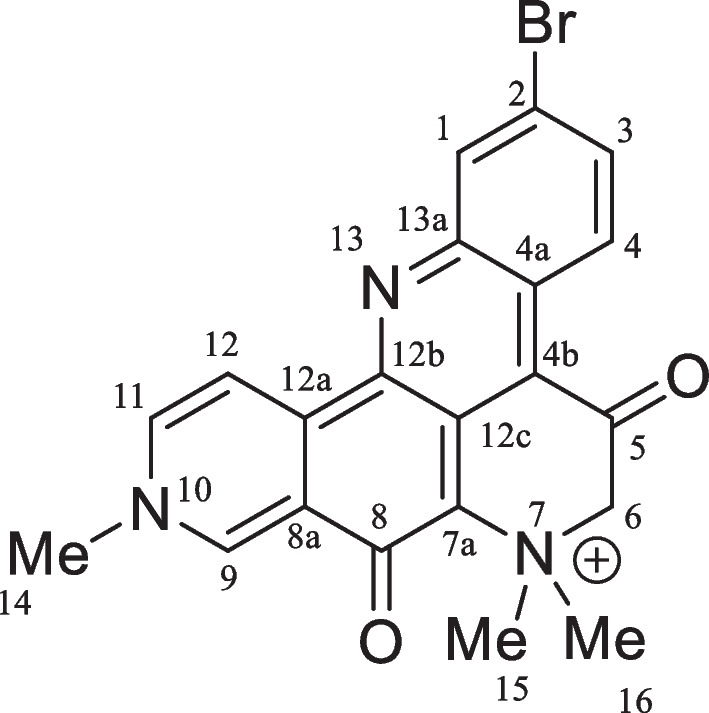
Chemical structure of petrosamine

The newly collected sponge was immersed in ethanol and stored in a freezer. Two portions of approximately 500 g of the sponge material were cut into small pieces and extracted with MeOH (2 × 600 mL) under an ultrasonication bath. The extracts were combined and the solvent evaporated at reduced pressure (40 °C) to approximately 200 mL of a hydromethanol fraction which was partitioned with n-hexane, CH_2_Cl_2_, and EtOAc (3 × 200 mL of each solvent). The resulting fractions were dried with anhydrous sodium sulfate (Na_2_SO_4_) and concentrated under reduced pressure to yield fractions n-hexane (1.4 g) CH_2_Cl_2_ (0.7 g), and EtOAc (0.2 g). The CH_2_Cl_2_ fraction was subjected to a Sephadex LH-20 column and eluted with a binary mixture of CH_2_Cl_2_/MeOH 2:8. A subfraction (213 mg) enriched in a deep blue material was purified by HPLC using a phenyl-hexyl semi-preparative column and a gradient solvent system of H_2_O (0.01% TFA)/MeOH 60–100% by 15 min, flow rate 3.0 mL/min, temperature 35 °C, to yield a pure compound (45.5 mg, t_*R*_ 10.6 min (Additional file 1: Fig. S1), which was unequivocally identified as petrosamine (Fig. 1) based on 1D and 2D NMR spectra.

#### Spectroscopic data

Dark blue needles: mp 330–332 °C; UV-PDA (H_2_O/MeOH) l_max_ 286, 345, 589 nm. ^1^H NMR (300 MHz in MeOD) *δ*_H_ (ppm): 9.80 (1H, s, H-9), 9.45 (1H, d,* J* = 5.7 Hz, H-12), 9.13 (1H, d, *J* = 9.0 Hz, H-4), 9.05 (1H, d, *J* = 5.7 Hz, H-11), 8.29 (1H, br s, Hz, H-1), 7.78 (1H, br d, *J* = 9.0 Hz, H-3), 4.63 (2H, s, 2H-6), 4.67 (3H, s, Me-14), 3.93 (6H, s, Me-15 and Me-16). ^13^C NMR (75 MHz in MeOD) *δ*_C_ (ppm): 188.1 (C-5), 162.5 (C-8), 146.7 (C-9), 144.7 (C-13a), 144.1 (C-12b), 143.2 (C-11), 141.2 (C-12a), 136.7 (C-3), 133.7 (C-8a and C-1), 130.1 (C-12c), 127.6 (C-4), 124.4 (C-4a), 123.9 (C-12), 122.6 (C-2), 117.2 (C-4b), 116.3 (C-7a), 72.0 (C-6), 54.7/54.5 (C-15 and C-16), 49.3 (C-14) (Additional file 1: Figs. S2, S3).

### Petrosamine reveals a good biocompatibility profile

Despite all the efforts from the scientific community, disease-modifying treatments for AD are not yet available [15]. This neurodegenerative disease is among the pathologies with the lowest rate of drug development success, with 99% of drug candidates being discontinued after showing no clinical benefit [16]. Therefore, since the approval of galantamine for the treatment of AD patients, the search for new anticholinesterase alkaloids has escalated [11]. Petrosamine, a pyridoacridine alkaloid, is a natural compound isolated from marine sponges shown to be 6 times more potent than galanthamine in inhibiting AChE [17], with a AChE IC50 0.091 µM, but not yet tested systematically in vitro or in vivo [10] for its safety profile. Petrosamine’s biocompatibility was hereby tested to verify the best suitable concentration from a range of 0.01 to 0.15 mg/ml, using: (1) cytotoxicity in neuroblastoma cell line (SH-SY5Y); (2) fish embryotoxicity assay (FET) with zebrafish; and (3) hemolysis assay.

Cell viability was evaluated in SH-SY5Y cells by resazurin after exposure to petrosamine for 24 h (Fig. 1a). The percentage of cell survival for each sample was expressed and normalized against the life control (100% viability). The rate of viability slightly increased only with the lowest concentration of petrosamine (0.01 mg/ml) and then decreased with increasing concentration of this compound, being slightly lower than the control but without statistically significance, which attests to petrosamine being non cytotoxic in all tested concentrations. Interestingly, as pyridoacridine alkaloids are considered DNA binding molecules, their cytotoxicity screenings have mainly been focused in identifying those that kill cancer cells and not as much to guarantee their lack of cytotoxicity, particularly in the CNS [18].

Using the zebrafish embryotoxicity assay, recommended by the OECD for evaluation of acute toxicity of chemicals (OECD test guideline (TG) 236), the survival rate of the zebrafish embryos was tested until 120 h post fertilization (h_pf_), as observed in Fig. 1b. The four tested concentrations of petrosamine were added to the water from 72 h_pf_ until 120 h_pf_, as the AlCl_3_ treatment was applied in the first 72 h of fish development [19]. The highest concentration of petrosamine was lethal to all embryos at 120 h_pf_, while the remaining concentrations supported a survival rate of 100% for all timepoints. Cesário et al*.*, evaluated the cytotoxicity of three main isolated compounds (0.1, 0.5, and 1.0 mg/mL) from the marine sponge *Aplysina fulva*, on adult zebrafish, and showed that those concentrations tested were non-toxic, which goes accordingly to our results [20].

In order to determine the effects of petrosamine in erythrocytes, as intravenous application the most probable route of its administration, the hemolysis assay was performed. The determination of the hemolytic properties of petrosamine, in vitro*,* is an important step for a preliminary evaluation of interaction with the membrane of red blood cells to determine their safety, as it permits to understand their propensity to interact with cell membrane causing lysis. Results shown in Fig. 1c are normalized for the death control consisting of Triton 10 × at 10% (v/v). Based on all the results regarding its safety profile, the chosen concentration of petrosamine for subsequent studies on its capacity to counteract the AlCl3-induced neurotoxicity was 0.05 mg/ml.

### Petrosamine counteracts aluminium-induced neurotoxicity in vitro

**Fig. 2:**
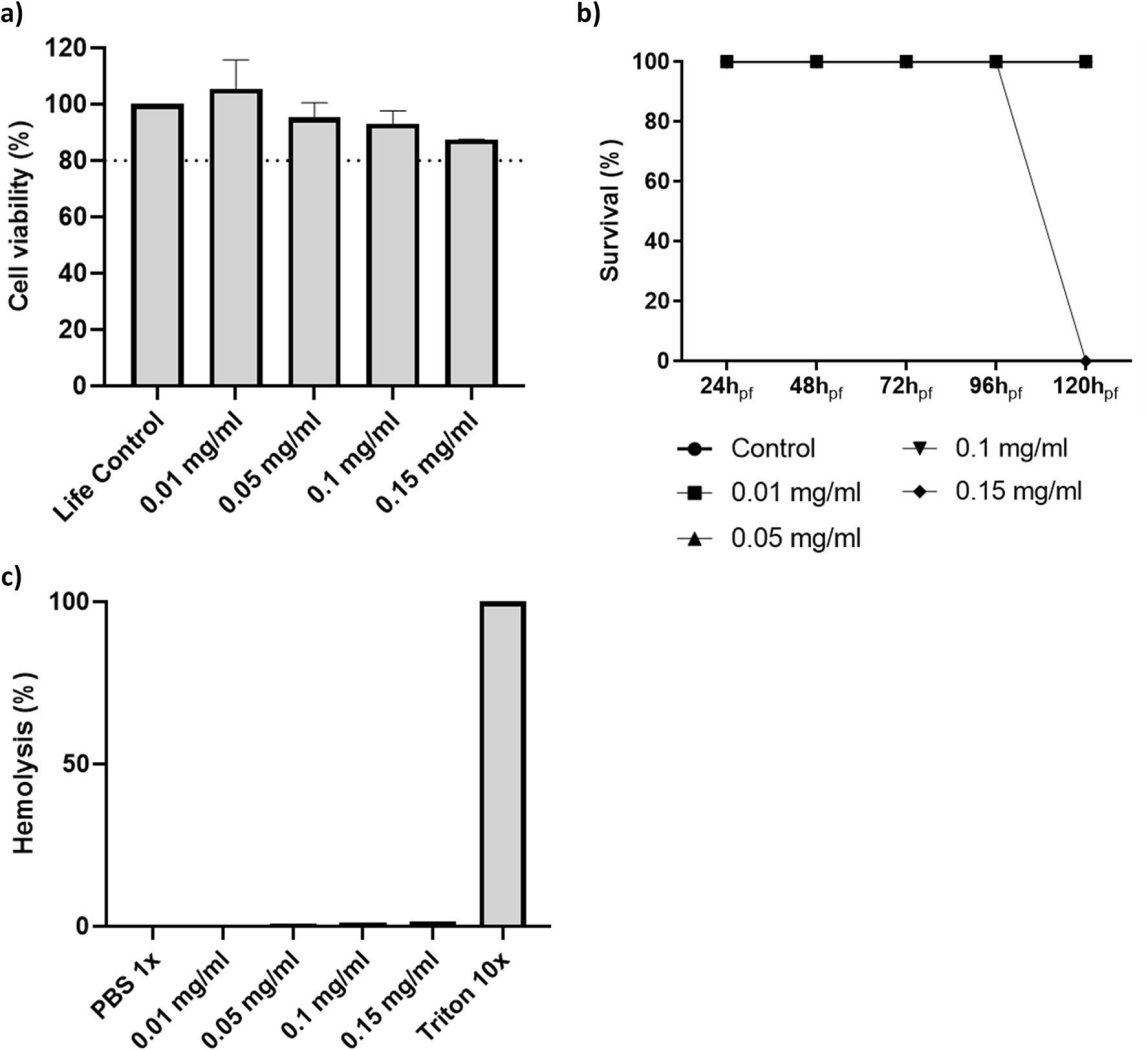
Biocompatibility of petrosamine. **a** Cytotoxicity of petrosamine in SH-SY5Y cell line after 24 h of incubation; **b** Survival rate of zebrafish embryos incubated with petrosamine from 72 to 120 hpf; **c** Evaluation of toxicity in erythrocytes incubated with petrosamine by hemolysis quantification

The scarce development of novel disease-modifying treatments and failed clinical trials are due to several factors, including premature translation of highly successful drugs in animal models that mirror only limited aspects of AD pathology to humans [21]. Consequently, there is a real necessity for model systems that can reproduce the pathological and biochemical characteristics of AD with the aim to allow adequate, fast preclinical screening of novel therapeutics [6]. In order to establish an AD model in vitro, cells from the neuroblastoma cell line SH-SY5Y were exposed to different concentrations of AlCl_3_ (50, 200 and 1000 µM) for 24 h. Metabolic viability was assessed with resazurin assay and the percentage of cell survival for each sample was expressed and normalized against the life control (100% viability). The resazurin assay showed that 50, 200 and 1000 µM petrosamine reduced cell viability by 14.0% (p < 0.05), 20.6% (p < 0.01) and 23.4% (p < 0.001), respectively (Fig. 2). In AD, there is a significant degree of apparent neuronal death in various areas of the brain [22] and AlCl_3_ properly reproduces this hallmark. A direct correlation between the increasing concentration of AlCl_3_ and the decrease in cell viability was observed. The concentration of 200 µM reduced cell viability to 79.3%, values very similar to those seen by Rizvi et al., that conducted a similar study (MTT assay) with 200 µM Al(mal)_3_ (74.5% cell viability) [23], p. 53]. Yang et al. have shown that the exposure of SH-SY5Y cells to aluminium ions in concentrations below 100 µM does not cause a significant reduction in cell viability and proliferation, which is consistent with the results obtained [24]. Thus, significant cell death could be observed at 1000 µM petrosamine, with 76.6% of cell viability.

Using this new in vitro model, results demonstrated that petrosamine (0.05 mg/ml or 0,01 mM) is efficient in restoring cell viability affected by 24 h exposure to AlCl_3_, since this natural compound could restore cell viability levels to those of the life control, when compared to cells incubated with AlCl_3_ only.

**Fig. 3:**
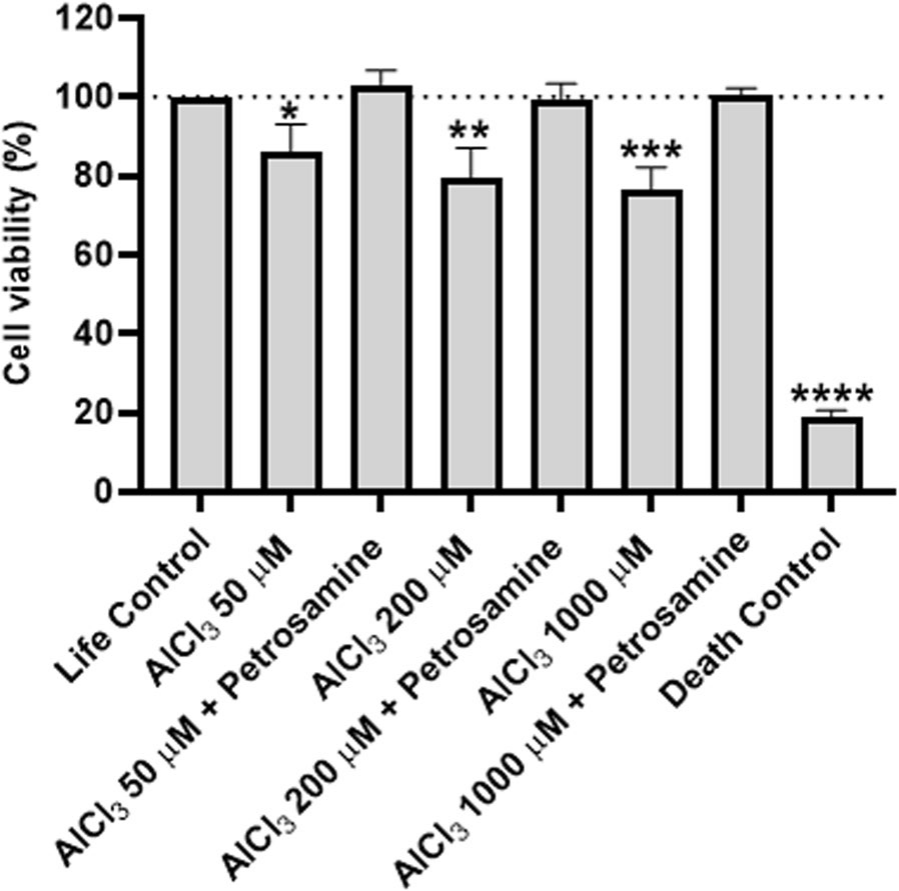
Evaluation of neuroprotection conferred by petrosamine in SH-SY5Y cell line exposed to AlCl_3_ for 24 h, measured by resazurin assay. Percentages are expressed relative to the life control (100% cell viability). Death control (30% DMSO). *p < 0.05, **p < 0.01, ***p < 0.001, ****p < 0.0001 when compared to the life control

Acridine orange (AO) / Propidium iodide (PI) assay was used to determine how AlCl_3_ induced neurotoxicity and to understand if the protection conferred by the petrosamine could translate into a reduction of apoptosis. While AO is a cell permeable green fluorescence probe, PI is a red fluorescent probe that only marks cells with compromised cell membranes and, consequently, stains only late apoptotic or necrotic cells. The double staining of the cells incubated with AlCl_3_ is presented in Fig. 3a to h. For this assay, two controls were performed, the life control (100% cell viability) and the death control (0% cell viability). In Fig. 3i, the percentage of viable cells, early apoptotic cells and late apoptotic/necrotic cells in each condition are reported. The number of cells allocated to each category was determined using ImageJ software. The AO/PI assay showed that 50, 200 and 1000 µM petrosamine led to 86.2% (p < 0.05), 79.8% (p < 0.01) and 57.3% (p < 0.0001) of viable cells, respectively. Thus, the number of early apoptotic and late apoptotic/necrotic cells increases with 24 h incubation with higher concentrations of AlCl_3_. As it is evident in Fig. 3, cells treated with petrosamine subsequently to AlCl_3_ incubation were mostly stained green, showing few apoptotic cells, while cells exposed to different concentrations of AlCl_3_ showed a significant portion of cells stained yellow and red, a marker of early apoptosis and apoptosis. In this assay, a reduction in apoptosis and concomitant restoration of cell viability are observed in the following conditions: 50 µM and petrosamine (95.8%), 200 µM and petrosamine (94.9%) and 1000 µM and petrosamine (94.0%).

Lipid peroxidation occurs when ROS react with susceptible unsaturated lipids on the cell membrane, potentially causing irreversible damage and it is known that Al leads to a higher production of ROS, stimulating iron-induced membrane lipid peroxidation [12]. To determine the lipid peroxidation level in SH-SY5Y cells, the TBARS assay was performed, and results of each sample were normalized against life control (100%). As shown in Additional file 1Fig. S1, no statistically significant differences were identified in lipid peroxidation of cells submitted to treatment with AlCl_3_, meaning that the tested concentrations do not have a substantial impact over this hallmark. Nevertheless, a clear trend is verified with the percentage of lipid peroxidation increasing with higher concentrations of AlCl_3_. Neurons are very sensitive to oxidative stress, which is the primary event in any neurological disorder [25]. Peroxidation of membrane lipids may be one of the mechanisms by which ROS contributes to the cascade of events leading to damage of cell membrane and also can induce peroxidation of myelin lipids [26]. When petrosamine is added to the cells, a reduction by approximately 5% in lipid peroxidation is verified in cells submitted to any AlCl_3_ concentration (Additional file 1: Fig. S4).

### Petrosamine potently inhibits AChE activity after aluminium-induced neurotoxicity in vitro

**Fig. 4:**
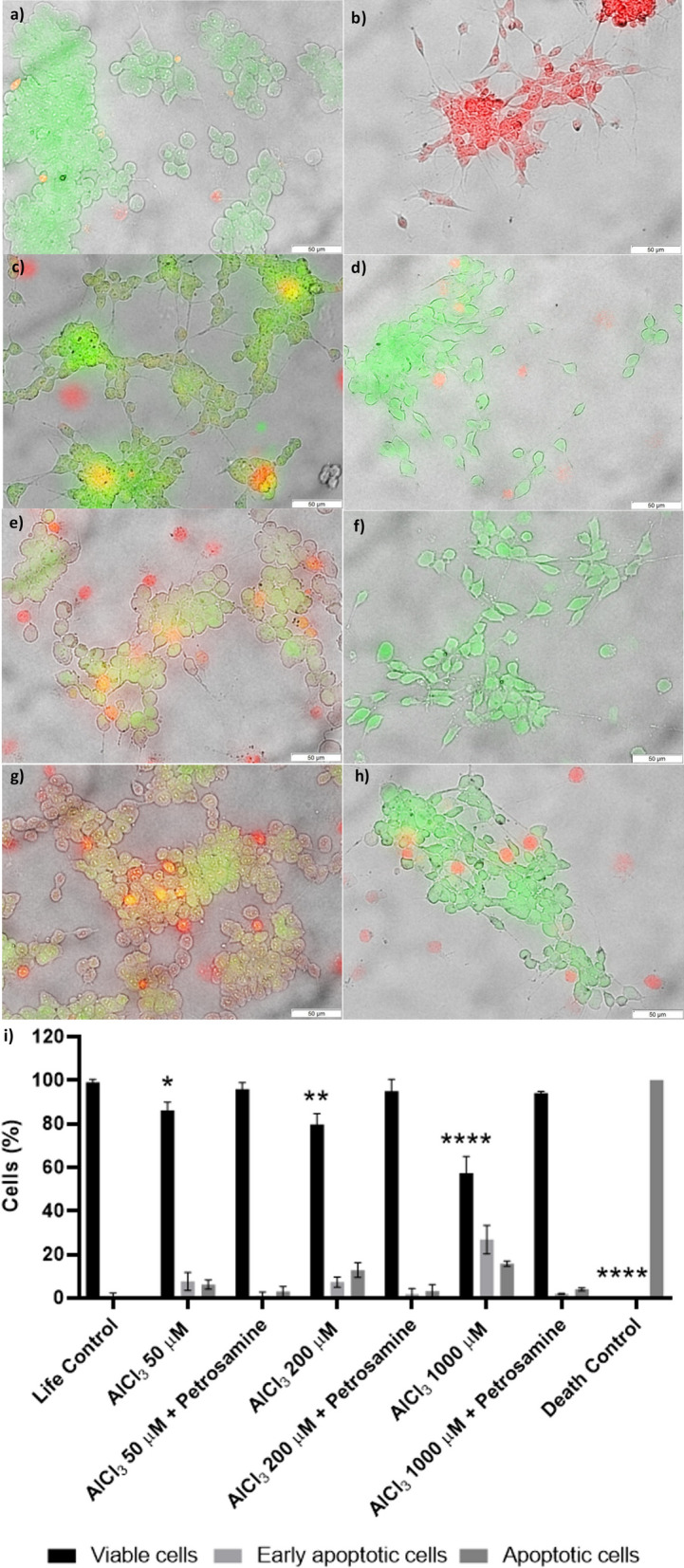
Fluorescence microscopy of acridine orange (AO)/propidium iodide (PI) assay showing SH-SY5Y cells exposed to different concentrations of AlCl_3_ for 24 h. **a** Life Control; **b** Death Control (30% DMSO); **c** 50 μM AlCl_3_; **d** 50 μM AlCl_3_ and petrosamine; **e** 200 μM AlCl_3_; **f** 200 μM AlCl_3_ and petrosamine; **g** 1000 μM AlCl_3_; **h** 1000 μM AlCl_3_ and petrosamine. Scale bar = 50 μm. Graph **i** represents the number of viable cells (green), early apoptotic cells (yellow), and late apoptotic/necrotic cells (red) in each condition. * p < 0.05, ** p < 0.01, **** p < 0.0001

The increase in AChE activity caused by different concentrations of AlCl_3_ applied to neuroblastoma cells (Fig. 4) was evaluated with Ellman’s colorimetric assay. AChE activity increased 48.9%, 195.7% (p < 0.05) and 314.6% (p < 0.001) upon treatment with 50, 200 and 1000 µM of AlCl_3_, respectively, in comparison to life control activity levels. One of the leading causes of the observed decline of the acetylcholine concentration in the brain of AD patients is due to the activity of AChE, which regulates the termination of the synaptic signal by hydrolysing the neurotransmitter acetylcholine secreted in the inter-synaptic cleft in the central nervous system [27]. AlCl_3_ appears to mimic AD-like neuropathology by altering AChE activity. This parameter has not yet been described in the literature to be affected in the neuroblastoma cell line SH-SY5Y, under the effect of AlCl_3_, validating the in vitro AD model used in this study. It is nevertheless acknowledged, in the literature, that Al can lead to a higher activity of AChE, decreasing the levels of acetylcholine release [28], which agrees with the results described here. To date, AChE inhibition has been the most widely used therapeutic target for symptomatic improvement in AD, since a cholinergic deficit is commonly observed in this disease [27]. As observed in Fig. 4, petrosamine leads to an intense decline of AChE activity to values lower than in the life control, possibly restoring the levels of acetylcholine neurotransmitter, after neurotoxicity induced in SH-SY5Y cells.

### Petrosamine can counteract most negative effects caused by AlCl_3_ on zebrafish development parameters

**Fig. 5:**
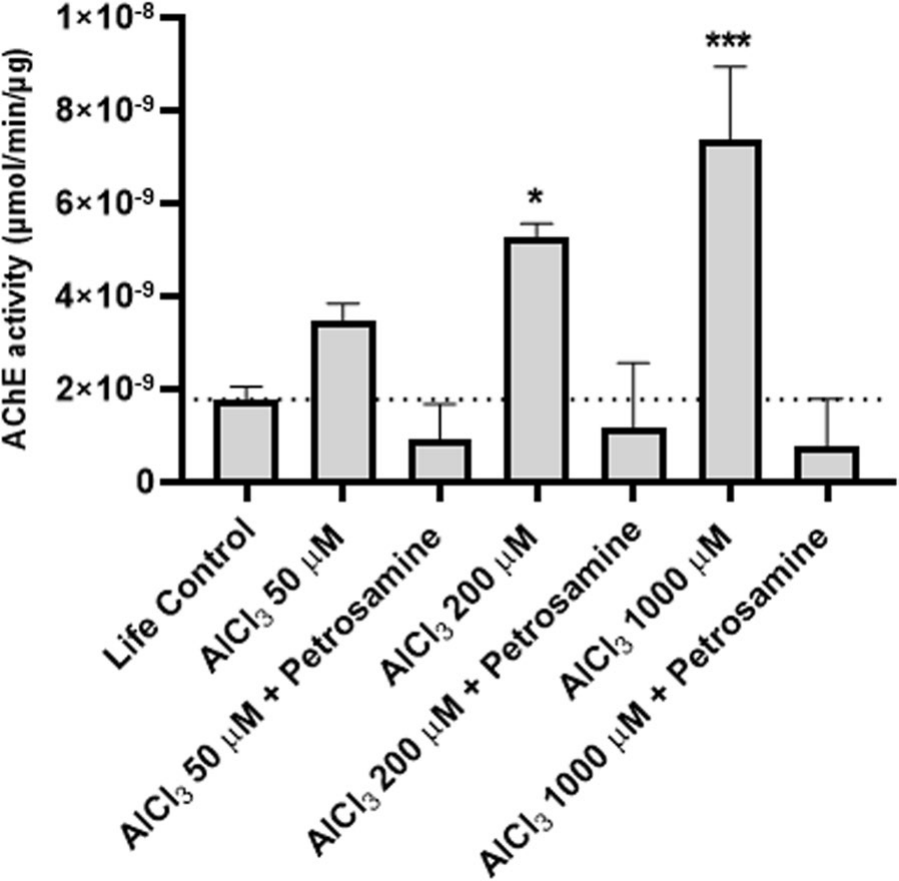
Activity of acetylcholinesterase (AChE) enzyme in SH-SY5Y cell line after 24 h of incubation with AlCl_3_ and further 24 h of petrosamine incubation. * p < 0.05 and *** p < 0.001 when compared to the life control

To evaluate in vivo the neurotoxicity provoked by AlCl_3_, several phenotypic parameters of zebrafish embryo development were assessed, including the survival and hatching rate, the yolk volume and the cardiac frequency. Zebrafish embryos were exposed to AlCl_3_ (50 and 200 µM) until 72 h_pf_ and then treated with petrosamine (0.05 mg/ml) until 120 h_pf_. The survival rate was evaluated daily during this time. As shown in Fig. 5a, the survival rate of embryos exposed to either concentration of AlCl_3_ decreased by 20% in the initial 24h_pf_ and addition of petrosamine did not influence this parameter. Zebrafish embryos typically hatch between 48 and 72 h_pf_ [29], so this parameter was only monitored up to 72 h_pf_ indicating the direct effect of exposure to Al. As shown in Fig. 5b, there is a trend of delayed hatching rate of zebrafish embryos exposed to AlCl_3_, in a dose-dependent manner, when compared to the control. Capriello et al*.* observed that Al presence led to hatching levels always lower than to the control and, surprisingly, this effect negatively correlated with AlCl_3_ concentration [30]. The toxicity mechanisms of Al are not sufficiently elucidated and these authors hypothesize that sublethal concentrations of Al differentially affect movement parameters and neuroblast differentiation, with only low levels of metal effecting alterations, thereby allowing extensive sublethal damage, while high levels of metal are capable of activating protective mechanisms.

**Fig. 6:**
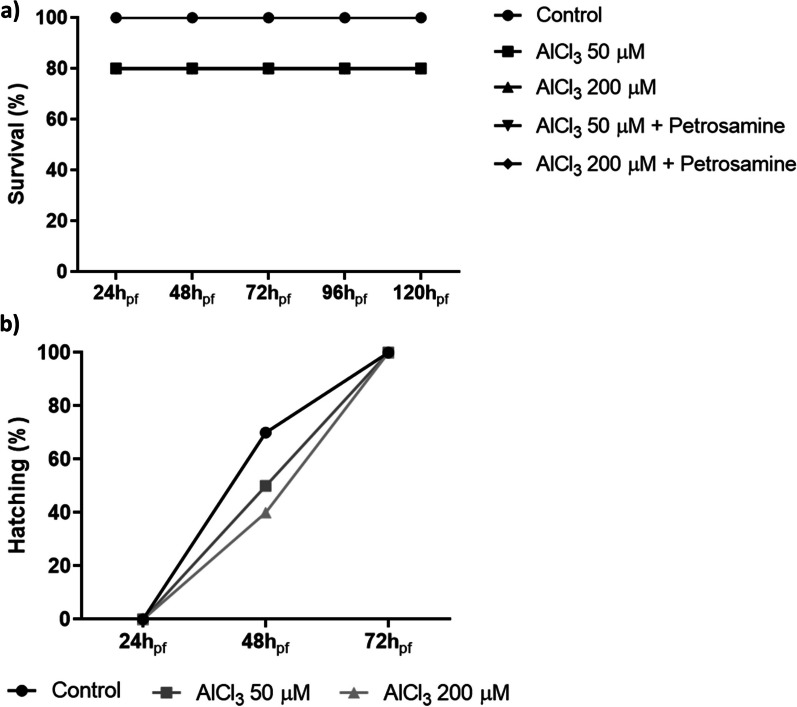
Analysis of zebrafish embryo development during embryotoxicity assay. **a** Survival rate after exposure to AlCl_3_ (50 and 200 μM), with or without treatment with petrosamine (0.05 mg/ml); **b** hatching rate of zebrafish embryos exposed to AlCl_3_ (50 and 200 μM)

Epiboly occurs during gastrulation and is the first morphogenetic cell movement during zebrafish embryogenesis [29]. In normal conditions, zebrafish embryo’s epiboly at 5.3 h_pf_ is roughly 50% [31]. The epiboly percentage of control embryos and embryos submitted to 50 and 200 µM of AlCl_3_ treatment was measured at 6 h_pf_ (Fig. 6). The control was at 75% of epiboly, while zebrafish embryos exposed to 50 and 200 µM concentrations were at 45% (p < 0.05) and 27% (p < 0.001) of epiboly, respectively. This striking difference indicates that the embryos exposed to AlCl_3_ had a slower development, already verified at 6h_pf_.

The zebrafish embryo relies solely on a finite amount of nutrient reserve present on the yolk, which presents an opportunity to gain insight if AlCl_3_ exposure compromises this phenotypic parameter [32]. Measurement of the yolk dimensions of individual zebrafish embryos over the first 120 h_pf_ can be used to track overall pace of embryo development. At 48 h_pf_, the yolk volume of the embryos treated with either conditions of AlCl_3_ was observed to be higher than the control (Additional file 1: Fig. S5). However, only at 120 hpf was identified a statistical significance for embryos submitted to treatment with 50 µM of AlCl_3_ (p < 0.01). This has not yet been described for Al-mediated toxicity, but Aldavood et al. verified that the yolk sac areas, at 72 hpf were significantly bigger in zebrafish embryos submitted to cadmium exposure as compared to age-matched controls [33]. The effect of AlCl_3_ on the yolk volume translates into a slower or impaired development of the zebrafish. If the yolk volume does not reduce overtime, the energetic needs during normal development are not being met. We observed that Al toxicity was more marked in larval compared to embryo stages. This probably occurs due to the absence of the chorion in larvae, which in the early stage of development exerts a good protection for the embryo and may somewhat reduce the exposure to the metal. When petrosamine (0.05 mg/ml or 0.01 mM) is added at 72 h_pf_, it limits this tendency in the yolk volume at 96 and 120 h_pf_, showing a great impact counteracting AlCl_3_-mediated toxicity and helping to accelerate the organism development.

The regular zebrafish heart rate ranges 140 to 180 beats per minute (BPM), considerably similar to the human fetal cardiac frequency (130–170 BPM) [34]. The exposure to AlCl_3_ greatly diminished the cardiac frequency and, as early as 24 h_pf_, zebrafish embryos exposed to AlCl_3_ exhibit a cardiac frequency lower than control embryos by approximately 50 BPM (Additional file 1: Fig. S6). There is usually a 20 to 50 BPM increase in heart rate associated with acquired swimming activity as early as day 5, suggesting that neural mechanisms involved in cardiac frequency acceleration are functional in early development [34]. This was not observed in zebrafish larvae exposed to AlCl_3_, suggesting compromised neural machinery that ultimately affects the cardiac function, through the parasympathetic and sympathetic branches of the autonomic nervous system [35]. The embryos also developed sublethal phenotypic alterations, such as pericardial edema (not shown). Monaco et al. also reported a reduction in cardiac frequency of zebrafish embryos treated with 100 µM of AlCl_3_ up to 48 h_pf_, in agreement with our results [19]. Similarly, Gouva et al. submitted zebrafish embryos to 0.05 mg/ml or 0,01 mM.Al treatment and registered a cardiac frequency around 144 BPM [36]. In our study, petrosamine was added at 72 h_pf_ but it did not significantly impact against Al-induced cardiotoxicity in zebrafish embryos (Additional file 1: Fig. S3).

### Petrosamine counteracts apoptosis induced by AlCl_3_ in zebrafish larvae

**Fig. 7:**
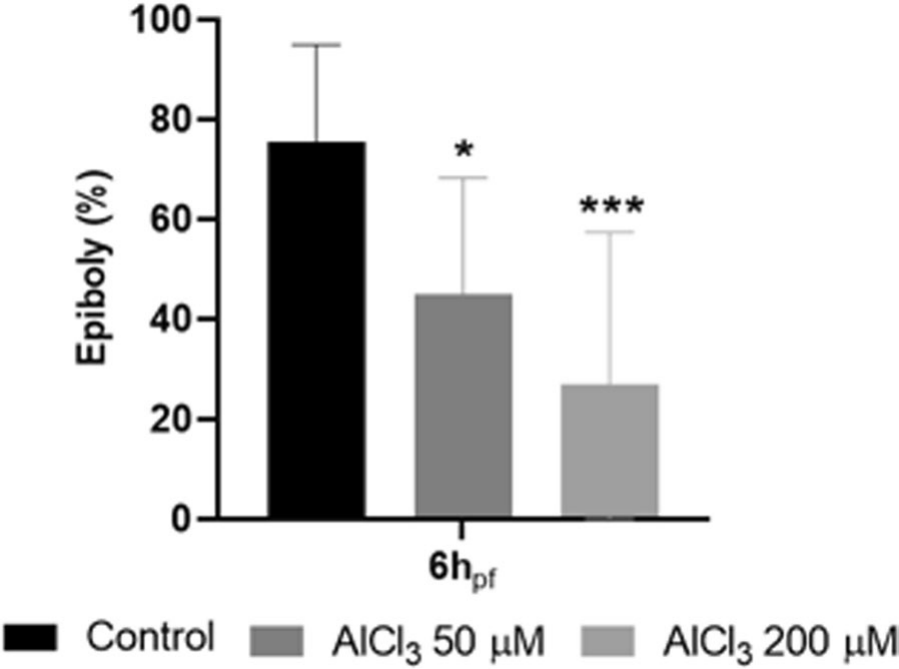
Epiboly of zebrafish embryos exposed to 50 and 200 µM of AlCl_3_ at 6 h_pf_. *p < 0.05 and ***p < 0.001 when compared to the control

Control larvae with a normal phenotype (Fig. 7a) and treated larvae with either 50 µM (Fig. 7b) or 200 µM (Fig. 7d) of AlCl_3_ were exposed to acridine orange to evaluate apoptosis by fluorescence microscopy. A physiological presence of apoptotic cells can hardly be observed in control larvae. However, a pattern of apoptotic spots (indicated by arrows) was observed in the head of the larvae with 120 hpf exposed to both concentrations of AlCl_3_. Interestingly, when larvae are treated with petrosamine (0.05 mg/ml or 0.01 mM) after neurotoxicity induced with AlCl_3_ 50 µM (Fig. 7c) and 200 µM (Fig. 7e), no apoptotic spots are observed. In order to perceive which concentration of AlCl_3_ was causing more apoptosis, the total fluorescence of each green spot was measured using ImageJ software. As it is verified in Fig. 7f, the 50 µM concentration of AlCl_3_ induces a higher level of physiological apoptotic spots in the larvae’s head, as seen by an extremely higher fluorescence intensity, compared to the control and to the concentration of 200 µM of AlCl_3_.

Apoptosis levels were studied in the zebrafish larvae, with acridine orange, and a mosaic of apoptotic cells in the larvae head was observed. The lateral line is a sensory system used by aquatic animals to detect water flow. This morphological structure is modulated by sensory organs and neuromasts [37]. Neuromasts are innervated by sensory neurons, situated in two ganglia, that leads to the anterior lateral line projected in the head and posterior lateral line along the body. Thus, neuromasts are clusters of superficial hair cells, allowing fish to sense mechanical changes in water, playing a crucial role in behaviours such as rheotaxis, predator avoidance and schooling [37]. The localization of apoptotic cells detected coincides with that of neuromasts, according to the observed pattern of green spots. Additionally, the total fluorescence intensity registered with 50 µM AlCl_3_ exposure is much stronger than with 200 µM AlCl_3_, which is similar to that of the control. This indicates that the larvae exposed to 50 µM of AlCl_3_ have several apoptotic cells in the neuromasts, which suggests that the detection of water movement in these 5-day old larvae may be ineffective. Capriello et al. also reached a similar conclusion, in a study in which they evaluated apoptosis levels in zebrafish embryos treated with AlCl_3_. The authors verified an increase in apoptosis in the head and tail in the larvae exposed to 50 µM AlCl_3_, while those exposed to higher doses exhibited a decrease in the number of apoptotic cells [30]. Also, they tested the larvae motility and concluded that motility drastically decreased in embryos exposed to 50 µM AlCl_3_ but tended to increase in larvae exposed to high concentrations [30]. This is in good agreement with our results, where 50 µM of AlCl_3_ lead to apoptosis in neuromasts.

### Petrosamine diminishes AChE activity after aluminium-induced neurotoxicity in vivo

**Fig. 8:**
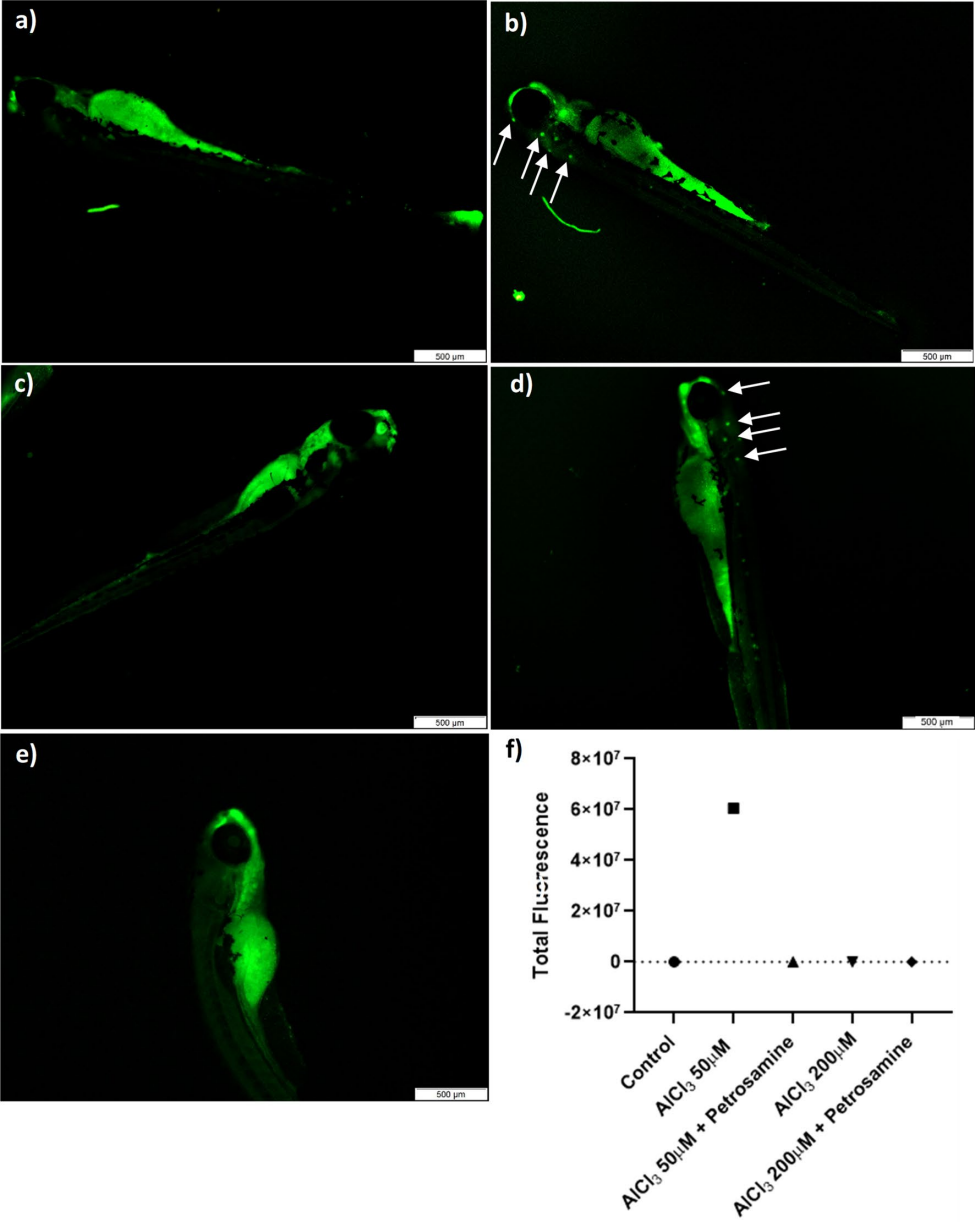
Acridine orange (AO) staining in whole body zebrafish larvae with 120 hpf.; **a** Control larvae; **b** larvae exposed to 50 μM AlCl_3_; **c** larvae exposed to 50 μM AlCl_3_ and petrosamine; **d** larvae exposed to 200 μM AlCl_3_; **e** larvae exposed to 200 μM AlCl_3_ and petrosamine; and **f** Total fluorescence of green spots indicating identified apoptotic cells. Particular fluorescent cellular accumulations are indicated by arrows. Scale bar = 500 μm

**Fig. 9:**
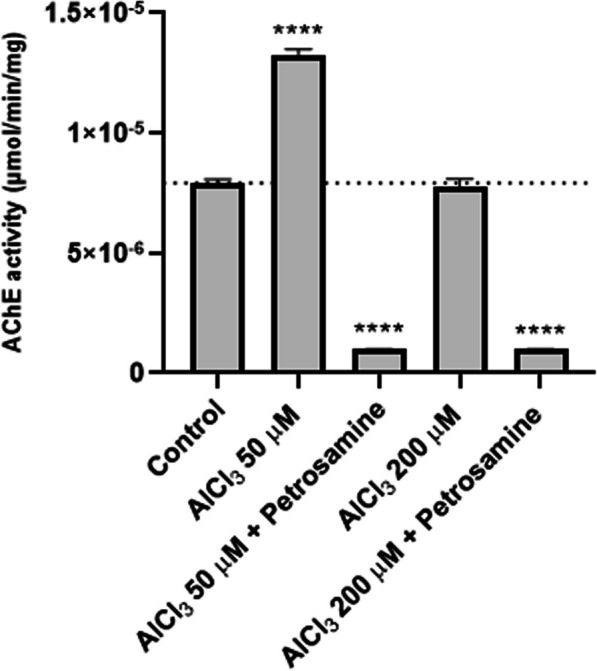
AChE activity of whole 120 hpf embryo, after exposure to two different concentrations of AlCl_3_ (50 µM and 200 µM) during 72 hpf and then treated with petrosamine (0.05 mg/ml) until 120 hpf. Statistical significance as compared to life control, ****p < 0.0001

Another harmful effect of Al on the nervous system of zebrafish embryos is the alteration of AChE activity, as observed in Fig. 8, also a hallmark in AD. In fish, AChE activity is essential for muscle function and behaviour [38]. This study examined the effects of AlCl_3_ exposure of AChE activity on whole zebrafish embryos (Fig. 8). Zebrafish embryos submitted to 50 µM of AlCl_3_ had an increased AChE activity by 67% (p < 0.0001). Senger et al*.* also verified a significant increase in AChE activity in the brain of adult zebrafish following exposure to AlCl_3_. The AlCl_3_ concentrations tested (50–250 μM) increased AChE activity by 28 to 33% [39], which is in line with our results. Senger et al. observed a smaller increase in AChE activity, since they targeted the enzyme only in the brain, excluding the remaining AChE present in the body. Oliveira et al. also reported a dose-dependent increase in enzymatic activity in adult fish *Oreochromis niloticus* after exposure to AlCl_3_ and Al_2_(SO_4_)_3_, measured both in individual brain and in individual muscle [40]. However, we did not verify an increase in the activity of this enzyme when the embryos were exposed to 200 µM of AlCl_3_. This occurred probably to the fact that the Al exposure was performed until 72 h_pf_, the embryonic stage, where the embryos are protected by the chorion, a biological structure surrounding the embryo until hatching to prevent toxins from entering in contact with the embryo [41]. Therefore, the ions in the highest concentration of AlCl_3_ may accumulate in the chorion, safeguarding the embryos from Al neurotoxicity. Petrosamine was applied at 72 h_pf_ after Al exposure leading to an AChE activity decrease by 92%, showing high AChE inhibition by this natural compound (Fig. 9).

Lin et al. tested the AChE inhibition of fucoxanthin, a natural carotenoid abundant in edible brown seaweeds, fucoxanthin significantly reversed the scopolamine-induced increase of AChE activity in the hippocampus and cortex of mice [42]. Additionally, Pam et al. tested derivatives of fascaplysin, an alkaloid isolated from a marine sponge *Fascaplysinopsis*, suggesting that one of the derivatives tested could penetrate the blood–brain barrier and be retained in the central nervous system and inhibit AChE with an IC50 value of 1 μM. Thus, Pam et al. verified that the fascaplysin derivative prevented cholinergic dysfunction and, consequently, prevented cognitive impairments by inhibiting AChE activity., in two AD mice models [43]. Nie et al. tested a marine fungal metabolite named butyrolactone I in vivo by implementing a cognitive deficit model in adult zebrafish, evoked by intraperitoneal injection of AlCl_3_, The authors verified that the AlCl_3_ treatment increased AChE activity, while butyrolactone I dose-dependently ameliorated AlCl_3_-induced cognitive deficits in zebrafish [44].

According to Nukoolkarn et al., petrosamine has an AChE IC50 of 0.091 µM [9], and the results obtained confirmed the great potential of this natural marine compound in the inhibition of this enzyme. Promptly focusing on AChE IC50, in 2000 the FDA has approved rivastigmine, a synthetic alkaloid and irreversible inhibitor of AChE, for the treatment of mild-to moderate AD, still used to date. Rivastigmine has an AChE IC50 5.5 µM [5, 45], while petrosamine has an AChE IC50 0.091 µM, being 60 × more effective in the inhibition of this enzyme, proving how petrosamine can be more efficient in AD treatment when compared with the two of the three anticholinesterase agents approved by the FDA (rivastigmine and galanthamine, as mentioned before). Stevensine has also been an alkaloid isolated from the sponge *Axinella verrucose*, collected from the Golf of Naples with an AChE IC50 12.2 µM [45, 46]. Thus, a steroidal alkaloid 4-acetoxy-plakinamine B, isolated from the marine sponge *Corticium* sp. had an AChE IC50 3.75 µM [47]. Besides alkaloids derived from marine sponges, several marine compounds also showed to have an AChE IC50 lower than petrosamine. An example is flustramine Q isolated from the bryozoan *Flustra foliacea* with an AChE IC50 of 9.6 µM [48] and Pulmonarin B isolated from the ascidian *Synoicum pulmonaria* with an AChE IC50 of 37.02 µM [45]. Petrosamine is not only one of the few marine-derived acetylcholinesterase-inhibiting alkaloid studied in vitro and in vivo that showed great features at cellular and molecular level against AD models but is also one compound with a potent AChE IC50, that can be recognized as for the fact of being one step forward for the development of new anti-AD drug.

## Conclusion

AD is a neurological disease that remains a public health challenge and still lacks effective treatments. One of the challenges of AD is to create models that best reproduce this pathology in order to evaluate promising disease-modifying therapeutics. AlCl_3_ showed great potential to induce AD-like toxicity in vitro and in vivo. Our study proved that exposure to Al affected some biological processes, including loss of neurons viability, lipid peroxidation, and an increase of AChE activity in the neuroblastoma cell line SH-SY5Y. The exposure of AlCl_3_ to the zebrafish embryos complemented the previous study, since we worked with a reliable animal model with several advantages, such as their CNS similarity with those of mammals, that allowed the better understanding of the AlCl_3_-induced neurotoxicity. Exposure to both concentrations of AlCl_3_ lead to cardiotoxicity, pericardial edema, development delays, apoptotic neuromasts, and increased AChE activity. The few reports in the literature, together with our promising results, suggest that there is a need for further experiments to elucidate the mechanisms underlying the Al-induced neurotoxicity.

Owing to a huge genetic diversity of organisms and an ecological and molecular diversity, oceans are a unique and rich source and reservoir of compounds with interesting biological activities that can be implemented in the treatment of various diseases. Many natural AChE inhibitors can be isolated from, marine bacteria, marine fungi, and especially marine sponges. A total of 185 marine cholinesterase inhibitors and selected analogs have been identified and reported and some of the compounds display inhibitory activities comparable or superior to cholinesterase inhibitors in clinical use. It is noteworthy that most of the marine compounds with inhibitor AChE properties studied to date, such as petrosamine, have demonstrated only in vitro AChE activity and very few of them have been tested on animal models, which is imperative to prove their capacity to cross the blood–brain barrier and exert beneficial effects in the brain. Petrosamine proved to be a safe, potent inhibitor of AChE against AlCl_3_-induced neurotoxicity in the neuroblastoma cell line SH-SY5Y and zebrafish embryos making this study a step forward in evaluating the translational value of this marine-derived alkaloid. Since the introduction of the galanthamine alkaloid as an antidementia drug in 2001, alkaloids have been one of the most attractive groups for searching for new AD drugs and more than two decades later the enhancement of the cholinergic neurotransmission still represents a main approach in the symptomatic treatment of AD. It is important to revisit anticholinesterase alkaloids that can act more efficiently towards AChE and in regard of their possible action in new pharmacological targets like neuroinflammation, amyloid beta, and tau protein.

## Experimental section

### Chemicals

Fetal bovine serum (FBS) and Penicillin/Streptomycin were purchased from Biochrom GmbH (Berlin, Germany), Dulbecco’s Modified Eagle’s Medium (DMEM) and AlCl_3_ were acquired from Sigma-Aldrich (St. Louis, MI). For the AlCl_3_ treatment, a stock solution of 10 mM dissolved in ultra-pure water was prepared. For the in vitro assays, AlCl_3_ was diluted with cell culture medium to obtain the final concentrations of 50, 200 and 1000 µM. For the in vivo treatments, AlCl_3_ was diluted with aquarium water, treated with UV light, to obtain final sub-lethal concentrations of 50 and 200 µM [19]. For the petrosamine treatment, a stock solution of 1 mg/ml was prepared dissolved in 3% DMSO. The final concentrations of 0.05 mg/ml were obtained by diluting the petrosamine in cell culture medium and aquarium water for in vitro and in vivo testing, respectively.

### Isolation and characterization of petrosamine

Chromatography columns were carried out on silica gel 60 (70–230 mesh, Merck) and Sephadex LH-20 (Pharmacia). while TLC was conducted on precoated silica gel aluminum sheets (60F254, 0.20 mm, Merck). The NMR spectra, in MeOD-d_4_, were run on a Bruker Avance DPX-300 spectrometer and the data were processed using the Academic TopSpin software by Bruker. A Shimadzu-UFLC semi-preparative HPLC system, equipped with ternary pumps and diode array SPD-M20A UV/VIS detector, was used for high-performance liquid chromatography (HPLC). The sponge *Petrosia* sp. was collected in March 2014, at the municipality of Trairí (3° 13′ 7,29″ S-39° 15′ 48,40″) in the state of Ceará, Brazil. A voucher specimen (MNRJ 17832) was deposited in the Porifera collection of the Museu Nacional, Universidade Federal do Rio de Janeiro, Rio de Janeiro, Brazil.

### Biocompatibility of petrosamine

Four concentrations of petrosamine (0.01, 0.05, 0.1 and 0.15 mg/ml) were evaluated in the neuroblastoma cell line SH-SY5Y, zebrafish embryos and in pig blood to assess its biocompatibility. The method used to evaluate the biocompatibility of this compound in the cells and in zebrafish embryos is forward described in this article. The hemolysis assay was completed according to Fernandes et al. [49]. In summary, the erythrocytes were obtained by several centrifugations at 4 °C under 600 *g*, for 5 min until the supernatant became transparent. Then, the erythrocytes sedimented in a pellet were resuspended in PBS 1 × . The different solutions of petrosamine were tested and the positive control was performed using only erythrocytes exposed to Triton 10x. The samples were incubated with the red blood cells at 37 °C for 30 min. After incubation, the samples were centrifuged at 4 °C under 600 *g*, for 5 min. The supernatant was collected, and the absorbance of hemoglobin was measured at 541 nm.

### In vitro neurotoxicity model

#### Cell culture and treatment

The establishment of the neurotoxic model and further evaluation of the petrosamine in vitro was assessed in the human neuroblastoma cell line SH-SY5Y. The cells were routinely grown in DMEM with phenol red supplemented with 15% (v/v) FBS, 1% (v/v) L-glutamine and 1% (v/v) Penicillin/Streptomycin and at 37° C in a humidified incubator with 5% CO_2_. The cells were subcultured in 25 cm^2^ flasks once a week. Cells were allowed to proliferate until they reached 80% confluence and then treated with 0.25% trypsin. The cells were plated in a 24-well plate at the density of 1 × 10^5^ cells/ml and in 96-well plate at the density of 2 × 10^4^ cells/ml. After overnight stabilization, the cells were treated with fresh medium containing 50, 200 and 1000 µM of AlCl_3_ for 24 h. On the following day, the AlCl_3_ treatment was removed and was added 0.05 mg/ml of petrosamine to each well. For all cell studies 3 replicate experiments were performed.

#### Cell survival assays

Cell viability was measured by resazurin reduction assay and by fluorescence microscopy using the Acridine orange (AO)/ Propidium iodide (PI) in 24-well plates. Cells treated with 30% DMSO served as a positive control for apoptosis. The resazurin assay was done according to Fernandes et al. [49]. Briefly, resazurin at 2.5 mM was added to each well for 2 h at 37 °C in the dark and the fluorescence intensity of the samples was measured at λ_Ex_ = 560 nm and λ_Em_ = 590 nm. For the AO/PI assay, 500 µl of PI (50 µM) were added to each well and the plate was left in the dark at 37º C for 15 min. Subsequently, another 500 µl of AO (50 µM) were added and the plate was again incubated in the dark at 37° C for 15 min. The fluorescence was visualized using FITC and TRITC filters in a fluorescence microscope (Olympus IX71). The percentage of viable cells, early apoptotic cells, and apoptotic cells in each condition was calculated using ImageJ software (Image Analysis Software, Rasband, NIH).

#### TBARS assay

The cells plated in the 96-well plate were lysed with Lysis Buffer (10 mM Tris, 1 mM EDTA, 0.2% Triton 100X). Each sample was collected to eppendorfs, and 1.5 ml of sodium phosphate buffer (pH = 7.4) were added, before incubating for 1 h, at 37 °C. Then, a volume 1:1 (v/v) of trichloroacetic acid (5%) and a 0.67% solution of Thiobarbituric acid (TBARS) (Sigma-Aldrich) were added to each sample, in 2:1 (v/v) proportion, and the samples were centrifuged at 2300 g for 15 min. The supernatant was collected and heated at 100° C for 15 min, and finally the absorbance was read at 532 nm in a microplate reader (Tecan, Infinite MPlex).

#### Determination of AChE activity in cells

The total protein was quantified with Bradford reagent using standard methodology. Ellman’s reaction is the most frequently used technique to assess cholinesterase activity [50]. The AChE detection mixture was prepared using acetylthiocholine iodide (1 mM) and 5–5’-dithiobis(2-nitrobenzoic acid) (DTNB) (0,5 mM), in a 1:1 volume. The assay medium was removed from the 96-well plate and 100 µl of the detection mixture was added to each well. AChE activity was measured spectrophotometrically, at 412 nm, in a microplate reader every minute for 60 min.

### In vitro neurotoxicity model

#### Zebrafish maintenance

Breeder adult zebrafish (*Danio rerio*) were housed in a properly oxygenated tank, at a temperature of 26 °C with a natural photoperiod of 12 h:12 h light/dark. The water quality parameters were checked regularly, and the fish were fed twice a day with a commercial diet (Tetra-Min).

#### Embryo treatment

The embryos laid at the first light in the morning were collected in clean aquarium water and selected with a stereomicroscope to eliminate unfertilized or unviable eggs. Assays were performed in non-treated 24-well plates with 10 embryos per well. The plates were incubated at 26 °C and 2 mL of test solutions were added to each well and renewed daily. Embryos were exposed to 50 and 200 µM of AlCl_3_ during the first 72 h post fertilization (hpf). At this time point, the AlCl_3_ solution was substituted by petrosamine (0.05 mg/ml) until 120 hpf. During the experiment, embryos exposed only to aquarium water, renewed every day, were used as control. All the treatments were applied up to 120 hpf to evaluate phenotypic parameters. At the end of the experiment, 10 larvae of each experimental group were used for apoptosis analysis by acridine orange staining. The remaining larvae with 120 hpf were euthanized in cold and stored at − 20 °C in RNA Later (Invitrogen), until further biochemical analysis.

#### Analysis of embryo development

Embryo development was monitored under Leica IL LED inverted microscope coupled with an ICC50 HD camera (Leica Biosystems, Germany) at a magnification of 4X. The survival rate was determined by counting the number of dead embryos at each time point and the hatching rate was checked until 72 hpf. Embryo development was documented at 24, 48, 72, 96, and 120 hpf, by imaging to determine the yolk volume in ImageJ and by video recordings to analyse the cardiac frequency.

#### Evaluation of apoptosis

After treatment, larvae with 120 hpf were moved to a new 24-well non-treated plate, rinsed with PBS 1 × and then leaved to incubate in 2 ml of AO (5 µg/ml) for 30 min in the dark, similar to the methodology of Capriello et al. [30]. Additionally, 0.4% of Tricaine was added for 15 min. Stained larvae were examined under fluorescent microscope, setting the FITC channel, emission peak at 525 nm. The total fluorescence of apoptotic spots in the head (green) was quantified using ImageJ software (Image Analysis Software, Rasband, NIH).

#### Determination of AChE activity

Larvae stored in RNA later − 20 °C were removed and washed in potassium phosphate buffer solution (KPBS). To evaluate AChE activity, 10 embryos per condition were homogenized in 800 µL of KPBS, by sonication during 10 s. The homogenates were centrifuged at 3000 *g* for 10 min, at 4° C. The total protein was quantified with Bradford reagent and the measurement of AChE activity was completed, according to Gravato et al. [51]. An AChE reaction buffer with 5,5'-Dithiobis-(2-Nitrobenzoic Acid) (DTNB) (4 mM) and acetylthiocholine iodide (0.075 M) was prepared and added to the samples in a 96-well plate reader. The absorbance was measured at 412 nm each 10 s during 3 min in a microplate reader (IndiaMART, Bio-Rad).

### Statistical analyses

The results were obtained from at least three independent experiments (n) and expressed as mean ± standard deviation (SD). All different statistical analyses were performed using GraphPad Prism version 8.3.4. for Windows, GraphPad Software, La Jolla California USA. Statistical significance of the results was determined using one-way ANOVA. Differences were considered statistically significant at P < 0.05.

### Supplementary Information

**Additional file 1: Figure S1.** HPLC chromatogram and PDA UV–Vis spectrum of petrosamine. **Figure S2.** ^1^H NMR (300 MHz, in MeOD) spectrum of petrosamine. **Figure S3.** ^13^C BB and APT NMR (75 MHz, in MeOD) spectra of petrosamine. **Figure S4**. Lipid peroxidation percentage after SH-SY5Y cells’ exposure to AlCl_3_ (50 µM, 200 µM and 1000 µM) for 24 h and then treated with petrosamine (c = 0.05 mg/ml) for 24 h. The results were expressed in relation to the life control (100%). No significant difference among means was verified. F**igure S5**. Yolk volume of zebrafish embryos at 24, 48, 72, 96 and 120 hpf. The embryos were submitted to (a) AlCl_3_ 50 µM and 200 µM the first 72 hpf and then treated with petrosamine until 120 hpf. ** p < 0.01 when compared to the control. F**igure S6**. Cardiac frequency of zebrafish embryos at 24, 48, 72, 96 and 120 hpf. The embryos were submitted to AlCl_3_ 50 µM and 200 µM the first 72 hpf and then treated with petrosamine until 120 hpf. **** p < 0.0001 when compared to the control.

## Data Availability

Data and/or material will be made available upon reasonable request.
