# Supplementary material for: Petrosamine isolated from marine sponge Petrosia sp. demonstrates protection against neurotoxicity in vitro and in vivo
Source: Nat Prod Bioprospect. 2024 Feb 22;14(1):16. doi: 10.1007/s13659-024-00439-x (PMC10881933; doi:10.1007/s13659-024-00439-x)
Supplement: Supplementary file 1 — Additional file 1: Figure S1. HPLC chromatogram and PDA UV–Vis spectrum of petrosamine. Figure S2. 1H NMR (300 MHz, in MeOD) spectrum of petrosamine. Figure S3. 13C BB and APT NMR (75 MHz, in MeOD) spectra of petrosamine. Figure S4. Lipid peroxidation percentage after SH-SY5Y cells’ exposure to AlCl3 (50 µM, 200 µM and 1000 µM) for 24 h and then treated with petrosamine (c = 0.05 mg/ml) for 24 h. The results were expressed in relation to the life control (100%). No significant difference among means was verified. Figure S5. Yolk volume of zebrafish embryos at 24, 48, 72, 96 and 120 hpf. The embryos were submitted to (a) AlCl3 50 µM and 200 µM the first 72 hpf and then treated with petrosamine until 120 hpf. ** p < 0.01 when compared to the control. Figure S6. Cardiac frequency of zebrafish embryos at 24, 48, 72, 96 and 120 hpf. The embryos were submitted to AlCl3 50 µM and 200 µM the first 72 hpf and then treated with petrosamine until 120 hpf. **** p < 0.0001 when compared to the control. [file 13659_2024_439_MOESM1_ESM.pdf]

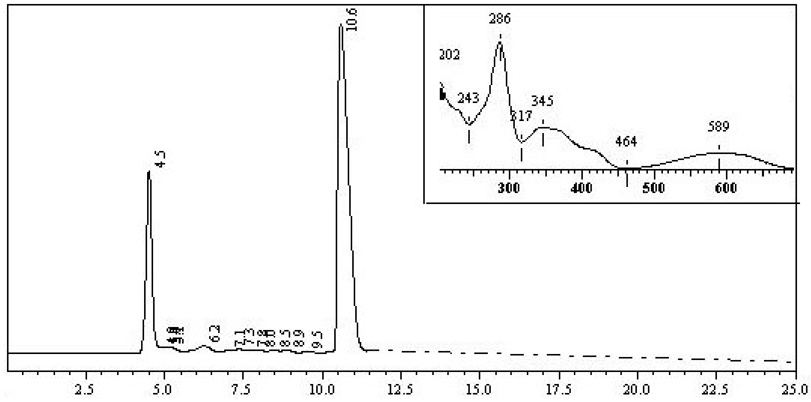

Supplementary Figure 1: HPLC chromatogram and PDA UV-Vis spectrum of petrosamine.

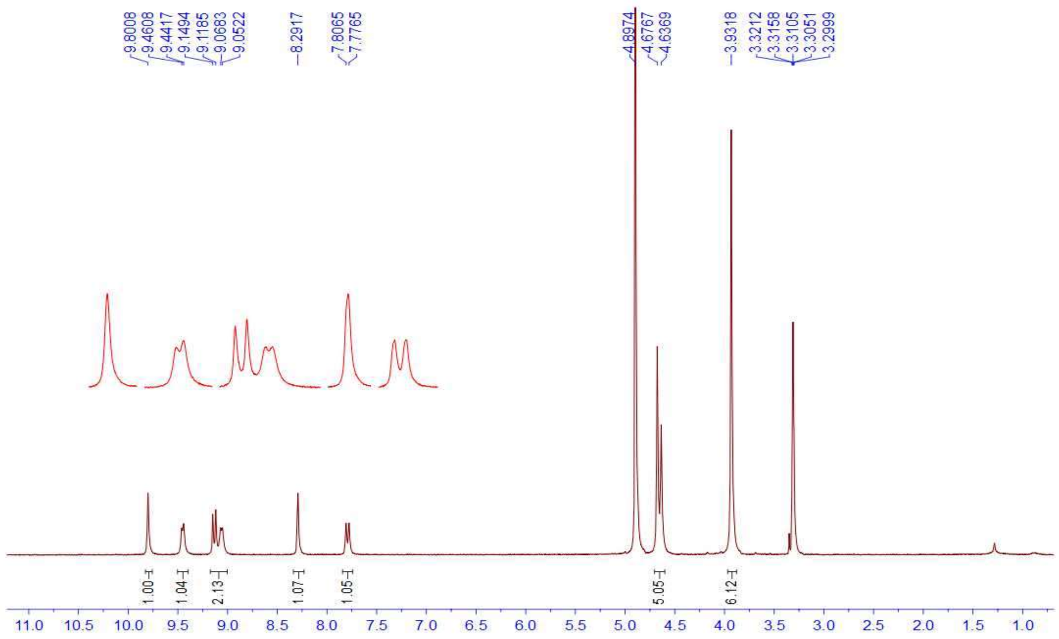

Supplementary Figure 2. ^{1}H NMR (300 MHz, in MeOD) spectrum of petrosamine

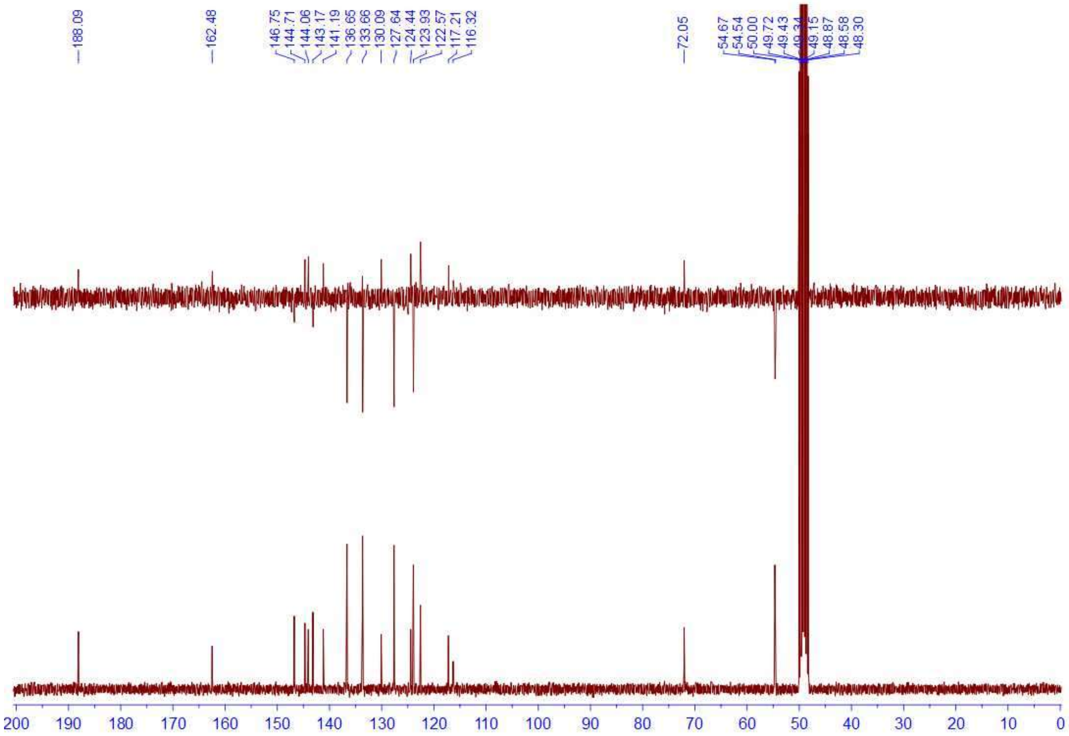

Supplementary Figure 3. ^{13}C BB and APT NMR (75 MHz, in MeOD) spectra of petrosamine

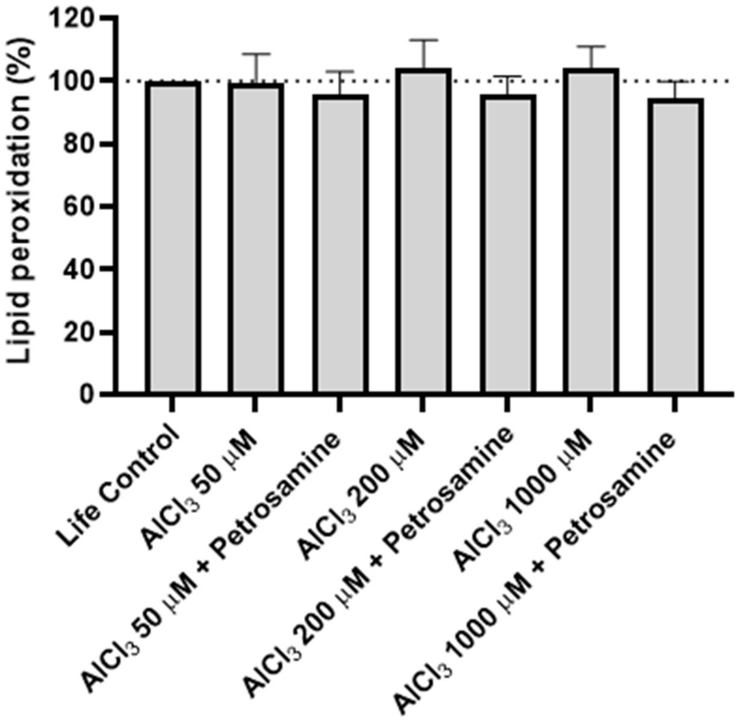

Supplementary Figure 4 . Lipid peroxidation percentage after SH-SY5Y cells' exposure to $AlCl_3$ (50 µM, 200 µM and 1000 µM) for 24h and then treated with petrosamine (c= 0.05 mg/ml) for 24h. The results were expressed in relation to the life control (100%). No significant difference among means was verified.

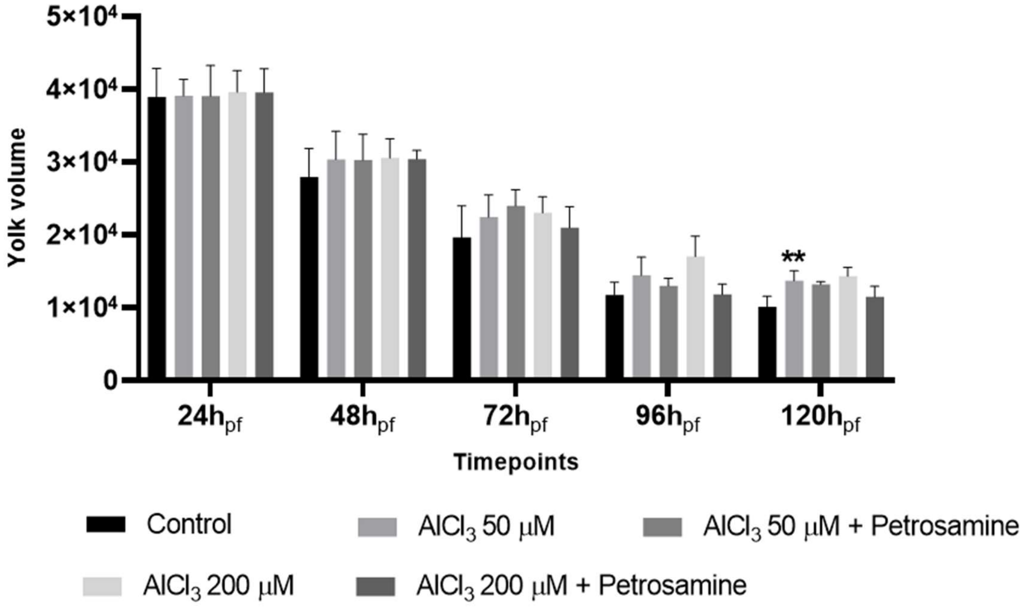

Supplementary figure 5. Yolk volume of zebrafish embryos at 24, 48, 72, 96 and 120 hpf. The embryos were submitted to (a) $AlCl_3$ 50 μM and 200 μM the first 72 hpf and then treated with petrosamine until 120 hpf. ** $p < 0.01$ when compared to the control.

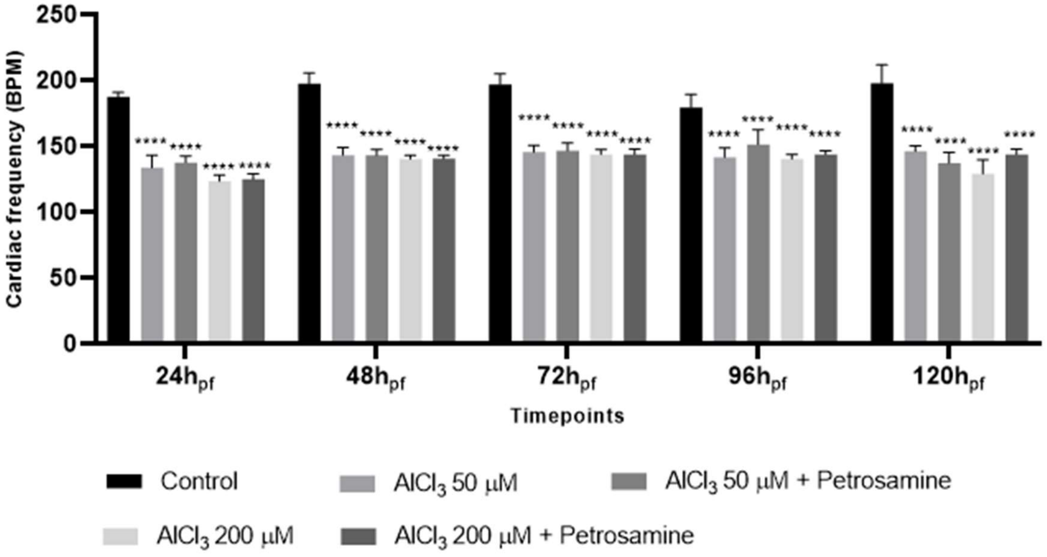

Supplementary figure 6. Cardiac frequency of zebrafish embryos at 24, 48, 72, 96 and 120 hpf. The embryos were submitted to $AlCl_3$ 50 μM and 200 μM the first 72 hpf and then treated with petrosamine until 120 hpf. **** $p < 0.0001$ when compared to the control.
